# Risperidone-Induced Hypothermia in a Cerebral Palsy Patient: A Case Report and Literature Review

**DOI:** 10.7759/cureus.76321

**Published:** 2024-12-24

**Authors:** Hayfaa T Taher, Thekra I Alsalmi, Alia M Alshalawi, Jehan F Sarriyah

**Affiliations:** 1 Internal Medicine, King Abdulaziz Specialist Hospital, Taif, SAU; 2 Medicine, Taif University, Taif, SAU

**Keywords:** bradycardia, cerebral palsy, post-cardiac arrest, risperidone-induced hypothermia, secondary epilepsy

## Abstract

Cerebral palsy (CP) is a debilitating disorder that can lead to life-long disability, with a high incidence in Saudi Arabia. Secondary epilepsy and cardiac complications are common in CP patients. We present a rare case of a 17-year-old female with CP, attention-deficit hyperactivity disorder (ADHD), secondary epilepsy, and a history of post-cardiac arrest, with home medications carbamazepine, risperidone, and sodium valproate. The patient presented with behavioral changes, bradycardia, hypothermia, and hypotension. The patient experienced a generalized tonic-clonic seizure, which was treated. Despite initial interventions, bradycardia and hypothermia persisted. Cardiology evaluation revealed normal cardiac function. Risperidone was temporarily replaced with clonazepam and hydrocortisone, resulting in the patient's arousal and stable vital signs. During the course of hospitalization, the patient also developed watery diarrhea, which was resolved after stopping antibiotics on the sixth day. The patient was discharged after 13 days with stable vital signs and follow-up instructions. This case highlights the complexity of managing CP patients with multiple comorbidities and the importance of a multidisciplinary approach to their care. It also underscores the urgent need for further research to improve the understanding of CP and its associated complications and develop more effective management strategies.

## Introduction

Cerebral palsy (CP) is a debilitating disease and is generally caused by damage to the brain of the fetus or infant. It is one of the most common disorders that can lead to life-long disability [1]. CP can cause a plethora of problems, ranging from spastic quadriplegia and spastic hemiplegia to the inability to perform voluntary movements and lack of muscle coordination to involuntary shaking [2]. The incidence of CP worldwide ranges between 2.3 and 3.6 per 1,000 live births [3]. However, its incidence in Saudi Arabia is 6.9 per 1,000 live births [4]. On the other hand, there are around 45 million people who are suffering from active epilepsy, which encompasses idiopathic and secondary epilepsy [5]. Secondary epilepsy is quite common in patients having CP, with a prevalence of 15-55% [6]. However, in children less than 18 years of age, the prevalence can be as high as 50%, even after antiseizure medication [7]. CP patients are sedentary due to problems with neuromuscular function, and this causes heart rate variability as well; these patients are more prone to cardiac diseases due to improper function of the autonomic nervous system, and this, in turn, can cause hypertension [8]. One review published by Amichai et al. in the pediatric CP population clearly shows that these children have a high variability in heart rate with overall higher heart rate due to dysregulation of the autonomic nervous system [9]. Herein, we present a unique and rare case of a 17-year-old female suffering from CP with a history of secondary epilepsy and previous post-cardiac arrest who presented in our clinic with behavioral changes. Further investigation pointed out that she had bradycardia and hypotension with normal O_2_ saturation. The patient did not respond to the initial treatment. However, medical management using midazolam, tazocin, and risperidone led to stabilizing the condition and discharge after 11 days of admission, following the resolution of hypothermia.

## Case presentation

We present the case of a 17-year-old Saudi female patient, a known case of CP, ADHD, and bedridden with secondary epilepsy that is currently treated using carbamazepine, risperidone, and sodium valproate. She also had a similar presentation two years ago when she had a cardiac arrest revived by CPR due to severe hypothermia. The patient presented to the emergency department (ED) with severe behavioral changes observed over the week before admission. Notably, the patient was found to be persistently cold by her mother the day before she arrived at the ED. While in the ED, the patient experienced a generalized tonic-clonic seizure, which was successfully terminated with midazolam.

Initial examination revealed a conscious but disoriented patient. Vital signs were notable for bradycardia (pulse: 55), hypotension (BP: 90/50), and hypothermia (temp: 32C). The patient had a blood oxygen saturation level (SpO_2_) of 96% on room air. The patient was admitted for hypothermia and started on tazocin 4.5 g IV every eight hours, sodium valproate 400 mg IV twice a day, carbamazepine IV three times a day, and risperidone 8 mL at bedtime. Despite initial interventions, the patient's sinus bradycardia (HR: 34) with normal respiration (RR: 20) and hypothermia (oral temp: 36.2°C) persisted into the first day of admission, prompting the use of a warming blanket and warm intravenous fluids. By the third day, the patient's vitals had stabilized, and she was transferred to a regular ward. However, the patient developed watery diarrhea by the sixth day, which was not associated with blood, mucus, abdominal pain, nausea, vomiting, or fever. Following a negative septic screen and unremarkable stool analysis, antibiotics were discontinued. Subsequently, the patient's diarrhea resolved. During the patient's hospital stay, her vital signs and platelet count were monitored daily (Table 1).

**Table 1:** Daily recorded temperature, pulse, blood pressure, and platelet count of the patient during hospital stay. Normal ranges: Temperature: 36.4-37.6°C; Blood Pressure: < 120/80 mmHg; Platelets: > 270,000 platelets per microliter

Day	Temperature	Pulse	Blood pressure	Platelets
0	32.7	55	90/50	90
1	36.2	34	91/49	71
2	36.5	77	122/70	88
3	36.9	87	110/66	95
4	36	78	101/69	88
5	35.9	62	118/61	87
6	35.8	64	118/64	77
7	32.9	46	90/60	69
8	36.5	68	110/70	138
9	35.3	74	127/53	73
10	35.5	68	129/62	67
11	36.1	64	108/58	112
12	36.4	75	90/62	
13	36	63	115/61	

**Figure 1:**
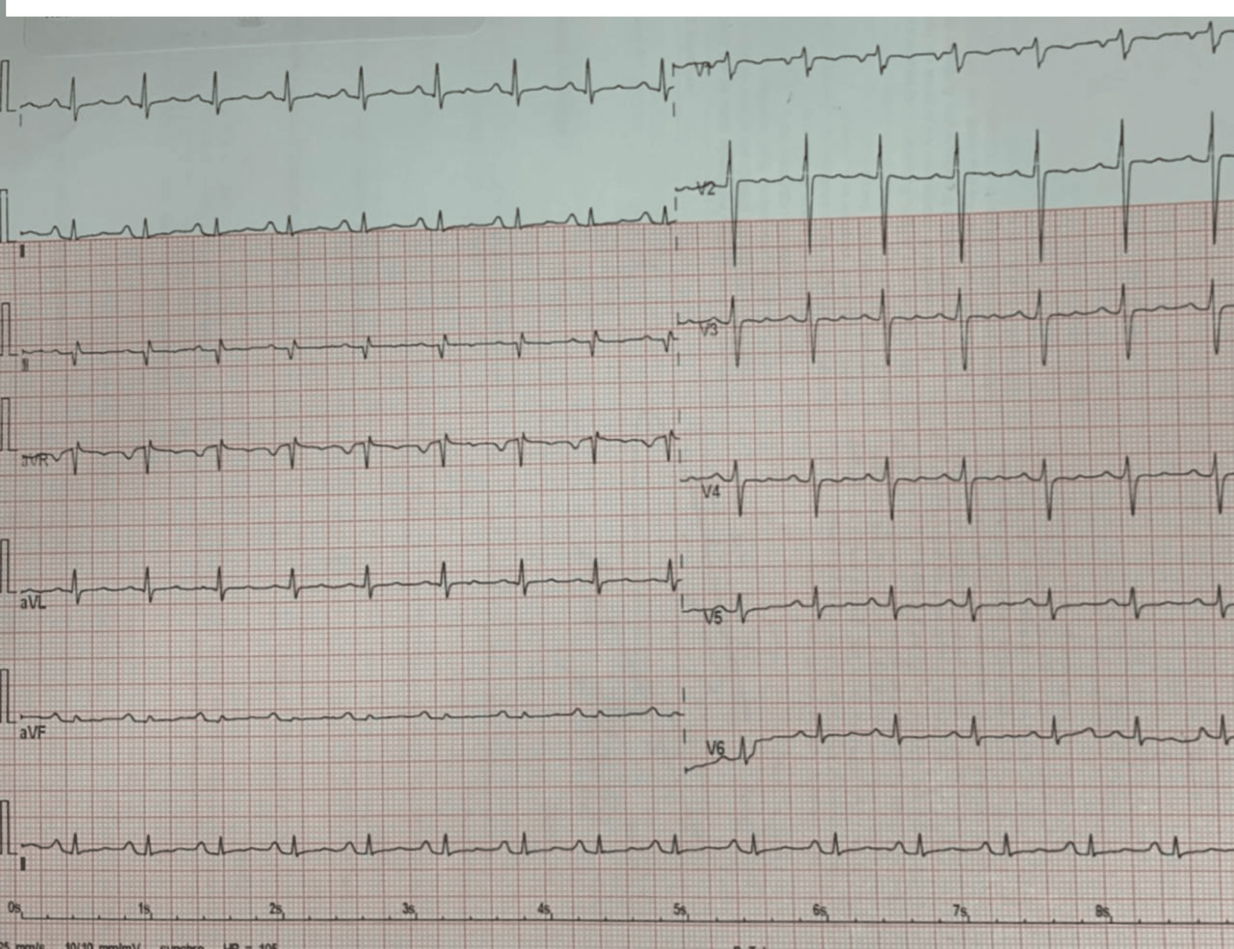
Electrocardiogram (ECG) of the patient on the seventh day of admission, showing a regular sinus rhythm with no abnormalities.

On the seventh day, the patient's bradycardia returned, and she was essentially unresponsive. Cardiology was consulted, and serial ECGs revealed a regular sinus rhythm (Figure 1). An echocardiogram showed normal cardiac chambers, an ejection fraction of 68%, no evidence of dysfunction, no thrombosis or masses noted, and normal pericardia with thin rim effusion. Clonazepam was temporarily discontinued in favor of hydrocortisone, which led to the patient's arousal and stable vital signs. However, on the 11th day, the patient showed signs of agitation, and clonazepam was resumed. After two more days of observation, the patient was discharged with stable vital signs and a temperature of 36.5 ℃. The discharge medications included carbamazepine syrup 200 mg twice daily, sodium valproate syrup 400 mg twice daily, and clonazepam 2.5 mg/mL, three drops once daily. The patient was evaluated by psychiatry and advised to discontinue risperidone for one month with outpatient department follow-up.

## Discussion

CP is one of the most prevalent and common causes of disability during childhood [10]. A plethora of risk factors are known to cause CP, including, but not limited to, low weight at birth, infection in the brain at birth, low oxygen supply, and abnormal development of the brain [7,11]. Diagnosis of CP is done during childhood and requires a multi-disciplinary approach; it includes birth history, investigation of posture along with motor functions, and neuroimaging [12]. Although CP may not be progressive, the symptoms may change with age [10]. The treatment strategy mainly focuses on physical rehabilitation and symptomatic treatment [13]. However, treatment is given symptomatically by a multi-disciplinary team of clinicians, including physicians from different specialties, nutritionists, therapists, and educational specialists [14,15]. Different medicines or interventions may be required to maintain or improve the quality of life of CP patients [10,16-18]. 

Risperidone is an important medication and is generally used for schizophrenia and maintenance of bipolar disorder [19]. Risperidone is commonly prescribed to CP patients to manage conditions such as irritability, agitation, ADHD, and involuntary movements such as dystonia or chorea, though it does not address spasticity directly [20]. Similarly, one study in 30 children with choreoathetoid CP found that treatment with risperidone for six months showed that the abnormal movements reduced significantly [21]. Risperidone has a good safety profile with only mild side effects, such as dizziness, nausea, weight gain, and sleep disturbances, even after long-term usage [22]. On the contrary, a case report of a physically healthy individual found that risperidone treatment caused thrombocytopenia and hypothermia [23]. Similarly, another study found risperidone in a child caused hypothermia [24]. This points to the fact that, although rare, risperidone may cause hypothermia in patients. Various mechanisms have been postulated. Risperidone has an affinity to serotonin receptors, which could affect thermoregulation, leading to hypothermia. Risperidone blocks alpha-2 adrenergic receptors to decrease vasoconstriction and shivering during cold, causing hypothermia. Risperidone’s affinity to serotonin receptors also affects platelet aggregation and causes thrombocytopenia. Hypothermia may develop a few weeks or years after treatment with risperidone, so regular monitoring of body temperature is essential for patients on antipsychotic medications [25].

In patients with CP, thermoregulation can be dysfunctional; thus, they generally have higher body temperatures owing to the increased requirement of energy for everyday tasks, as well as improper thermoregulation [7,11]. One study by Rossetti et al. found that 33% of the patients with CP had higher body temperatures and perspiration during sleep [26]. Due to this, hypothermia has been used as a treatment option in neonates who are born with signs and symptoms of encephalopathy to prevent not only CP later in life but also other neurological disorders [27,28]. One retrospective study compared the incidence of mild versus severe CP outcomes in neonates having intrapartum asphyxia treated with hypothermia. This study found that only 8% of the neonates developed CP [29]. Additionally, refractory status epilepticus (RSE) is a common manifestation of CP [17]. Several pharmacological and non-pharmacological options are used to control the RSE, which include drugs such as ketamine and sodium valproate and non-pharmacological treatment options, such as transcranial magnetic stimulations and hypothermia [26,30]. Similarly, another study found that, during exercise, CP patients were unable to properly lose heat from the body compared to the healthy population [31]. These studies point to the fact that CP can be prevented or at least treated in part with hypothermia [26-28,30,31]. What makes our study unique is the fact that, during admission, our patient was suffering from hypothermia, which is an atypical presentation of CP and rarely seen in clinical practice. Similar to our study, one case report showed that a CP patient was suffering from hypothermia during admission [32]. These point to the fact that, although hypothermia can be used as a therapeutic option, some CP patients may present with hypothermia. Thus, it is important to undertake more research to understand these atypical presentations of CP.

Patients with CP are known to have a higher incidence of hypertension, myocardial infarction, hyperlipidemia, and asthma, with an odds ratio of musculoskeletal at 6.97 and that of cardiometabolic morbidity at 1.98 [33]. In concurrence with this study, our study also found that the patient had a myocardial infarction two years before admission. Hypertension and myocardial infarction may be high in these patients due to early neurological damage, as well as a sedentary lifestyle [34].

Hypotension and heart rate variability are also common symptoms found in patients with CP [7,35]. One study found that 20% of the children suffering from CP had postural hypotension [35]. A systematic review of 12 studies found that tachycardia was prevalent, and heart rate variability was high in patients with CP [34]. In contrast, we found that our patient had bradycardia during the time of admission. These differences, in part, may be explained by CP patients having high heart rate variability, which may lead to either bradycardia or tachycardia.

An echocardiogram is a non-invasive technique that can detect several problems in the heart [36]. Due to the high rate variability, the sedentary lifestyle of CP patients, and the high risk of cardiometabolic disorders, the electrocardiogram is a valuable tool for detecting any heart abnormalities [33]. One study in pediatric CP patients found a higher heart rate and abnormal echocardiogram, which is short PR interval, shorter QRS, and short T-wave axis [37]. Although we did not find abnormalities in cardiac chambers in our patient, the patient had an ejection fraction of 68% with no evidence of dysfunction or thrombosis. This heterogeneity in disease presentation and causation may be due to the heterogeneous presentation of CP [38].

To our knowledge, this is the first case report of atypical CP presenting with hypothermia, bradycardia, and hypotension in a patient with a history of epilepsy, post-cardiac arrest, and ADHD on carbamazepine, risperidone, and sodium valproate, despite normal heart function. Hypothermia in CP may represent an area where more research needs to be done to understand the disease, which may help in better treatment options in the future. However, our study has a few limitations, such as it cannot be generalized to a large population, causal inference cannot be drawn, results cannot be overinterpreted, and there is publication bias.

## Conclusions

This case highlights an atypical presentation of cerebral palsy involving temperature dysregulation, bradycardia, and hypotension, potentially linked to antipsychotic use. The successful clinical management involved discontinuing risperidone and implementing supportive care measures, including hydrocortisone and careful adjustment of medications. This report underscores the importance of considering rare side effects of commonly used medications in cerebral palsy patients, emphasizing a multidisciplinary approach to managing complex clinical presentations.

## References

[1] Hoei-Hansen CE, Weber L, Johansen M (2023). Cerebral palsy - early diagnosis and intervention trial: protocol for the prospective multicentre CP-EDIT study with focus on diagnosis, prognostic factors, and intervention. BMC Pediatr.

[2] Hallman-Cooper JL, Rocha Cabrero F (2025). Cerebral palsy. StatPearls.

[3] McIntyre S, Goldsmith S, Webb A (2022). Global prevalence of cerebral palsy: a systematic analysis. Dev Med Child Neurol.

[4] Mushta SM, Alghamdi R, Almalki H (2024). Saudi cerebral palsy register (SCPR): protocol on the methods and technical details. J Epidemiol Glob Health.

[5] Beghi E, Giussani G, Nichols E (2019). Global, regional, and national burden of epilepsy, 1990-2016: a systematic analysis for the Global Burden of Disease study 2016. Lancet Neurol.

[6] Dos Santos Rufino A, Påhlman M, Olsson I, Himmelmann K (2023). Characteristics and challenges of epilepsy in children with cerebral palsy—a population-based study. J Clin Med.

[7] Gong C, Liu A, Lian B (2023). Prevalence and related factors of epilepsy in children and adolescents with cerebral palsy: a systematic review and meta-analysis. Front Pediatr.

[8] Ryan JM, Crowley VE, Hensey O, Broderick JM, McGahey A, Gormley J (2014). Habitual physical activity and cardiometabolic risk factors in adults with cerebral palsy. Res Dev Disabil.

[9] Amichai T, Katz-Leurer M (2014). Heart rate variability in children with cerebral palsy: review of the literature and meta-analysis. NeuroRehabilitation.

[10] Patel DR, Neelakantan M, Pandher K, Merrick J (2020). Cerebral palsy in children: a clinical overview. Transl Pediatr.

[11] Abd Elmagid DS, Magdy H (2021). Evaluation of risk factors for cerebral palsy. Egypt J Neurol Psychiatry Neurosurg.

[12] Jiang L, Yang W, Chen H, Song H, Zhang S (2024). Diagnosis and therapies for patients with cerebral palsy over the past 30 years: a bibliometric analysis. Front Neurol.

[13] Paul S, Nahar A, Bhagawati M, Kunwar AJ (2022). A review on recent advances of cerebral palsy. Oxid Med Cell Longev.

[14] Alrasheed A, Altulahi N, Temsah MH (2021). Interprofessional education competition during the COVID-19 pandemic at King Saud University: benefits and challenges. J Multidiscip Healthc.

[15] Novak I, Morgan C, Adde L (2017). Early, accurate diagnosis and early intervention in cerebral palsy: advances in diagnosis and treatment. JAMA Pediatr.

[16] Nahm NJ, Graham HK, Gormley ME Jr, Georgiadis AG (2018). Management of hypertonia in cerebral palsy. Curr Opin Pediatr.

[17] Pavone P, Gulizia C, Le Pira A (2020). Cerebral palsy and epilepsy in children: clinical perspectives on a common comorbidity. Children (Basel).

[18] Park TS, Dobbs MB, Cho J (2018). Evidence supporting selective dorsal rhizotomy for treatment of spastic cerebral palsy. Cureus.

[19] McNeil SE, Gibbons JR, Cogburn M (2023). Risperidone. StatPearls.

[20] da Silva JF, Honorato MM, Cremaschi RM, Coelho FM (2023). Efficacy and tolerance profile of risperidone use in people with autism spectrum disorder in a clinic in Santarém, Pará, Brazil. A retrospective study. J Neurosci Rural Pract.

[21] Kamate M, Mittal N, Metgud D (2018). Effect of risperidone on the motor and functional disability in children with choreoathetoid cerebral palsy. Pediatr Neurol.

[22] Perera MA, Yogaratnam J (2014). De novo delayed onset hypothermia secondary to therapeutic doses of risperidone in bipolar affective disorder. Ther Adv Psychopharmacol.

[23] Goyal L, Acebo R, Islam S, Ajmera K, Kaasam S (2022). A rare case of risperidone-induced hypothermia and thrombocytopenia. Cureus.

[24] Grau K, Plener PL, Gahr M, Denzer C, Freudenmann RW (2017). Mild hypothermia in a child with low-dose risperidone. Z Kinder Jugendpsychiatr Psychother.

[25] Ali W, Ahmad T, Ahmad K, Ali A (2023). Risperidone induced hypothermia and thrombocytopenia. Am J Respir Crit Care Med.

[26] Rossetti AO, Lowenstein DH (2011). Management of refractory status epilepticus in adults: still more questions than answers. Lancet Neurol.

[27] Gagne-Loranger M, Sheppard M, Ali N, Saint-Martin C, Wintermark P (2016). Newborns referred for therapeutic hypothermia: association between initial degree of encephalopathy and severity of brain injury (what about the newborns with mild encephalopathy on admission?). Am J Perinatol.

[28] Papile LA, Baley JE, Benitz W (2014). Hypothermia and neonatal encephalopathy. Pediatrics.

[29] Pekeles H, Al Amrani F, Perez-Morgui M, Wintermark P, Shevell M (2023). Characteristics of children with cerebral palsy in the post-therapeutic hypothermia era. J Child Neurol.

[30] Rossetti AO (2012). Hypothermia in refractory status epilepticus. Crit Care.

[31] Maltais D, Wilk B, Unnithan V, Bar-Or O (2001). Thermoregulatory resposes during exercise in a hot climate in children with spastic cerebral palsy. Med Sci Sports Exerc.

[32] Watters M, Wilson H, Everitt P (2019). Phenytoin-induced hypothermia. BMJ Case Rep.

[33] Whitney DG, Hurvitz EA, Ryan JM (2018). Noncommunicable disease and multimorbidity in young adults with cerebral palsy. Clin Epidemiol.

[34] Gąsior JS, Zamunér AR, Silva LE (2020). Heart rate variability in children and adolescents with cerebral palsy—a systematic literature review. J Clin Med.

[35] Azouz HG, AbdelMohsen AM, Abdel Ghany HM, Mohamed RM (2021). Evaluation of autonomic nervous system in children with spastic cerebral palsy: clinical and electophysiological study. Egypt Rheumatol Rehabil.

[36] Lee SH, Park JH (2023). The role of echocardiography in evaluating cardiovascular diseases in patients with diabetes mellitus. Diabetes Metab J.

[37] Pastore CA, Samesima N, Imada R (2011). Characterization of the electrocardiographic pattern of individuals with cerebral palsy. J Electrocardiol.

[38] Sadowska M, Sarecka-Hujar B, Kopyta I (2020). Cerebral palsy: current opinions on definition, epidemiology, risk factors, classification and treatment options. Neuropsychiatr Dis Treat.

